# Blood hemoglobin A1c levels and amyotrophic lateral sclerosis survival

**DOI:** 10.1186/s13024-017-0211-y

**Published:** 2017-09-21

**Authors:** Qian-Qian Wei, Yongping Chen, Bei Cao, Ru Wei Ou, Lingyu Zhang, Yanbing Hou, Xiang Gao, Huifang Shang

**Affiliations:** 1 Department of Neurology, West China Hospital, Sichuan University, 37 Guoxue Xiang, Chengdu, Sichuan 610041 China; 2 Department of Nutritional Science, The Pennsylvania State University, 109 Chandlee Lab, University Park, PA 16802 USA

**Keywords:** Amyotrophic lateral sclerosis, HbA1c, Fasting glucose, Survival, Body mass index

## Abstract

**Background:**

There are inconsistences regarding the correlation between diabetes or fasting blood glucose concentrations and the risk and survival of amyotrophic lateral sclerosis (ALS) in the previous studies. Moreover, the association between hemoglobin A1c (HbA1c) levels, which reflect long-term glycemic status, and ALS survival was not examined.

**Methods:**

A prospective cohort study including 450 Chinese sporadic ALS patients (254 men and 196 women; mean age: 55.4 y). We identified 223 deaths during average 1.6 years of follow-up. We assessed levels of fasting HbA1c (primary exposure) and glucose (secondary exposure) via ion exchange high-performance liquid chromatography and hexokinase/glucose-6-pgosphate dehydrogenase methods, respectively. Multivariate Cox proportional hazards regression model was used to calculate hazard ratios (HRs) and 95% confidence intervals (CIs) of ALS mortality across the exposures.

**Results:**

Our results indicated that, higher levels of HbA1c, but not fasting blood glucose concentrations, were significantly associated with higher risks of mortality. The adjusted HR was 1.40 (95% confidence interval (95% CI): 1.02–1.99) for HbA1c of 5.7–6.4%, and 2.06 (95% CI: 1.07–3.96) for HbA1c ≥6.5%, relative to HbA1c <5.7% (P trend =0.01), after adjustment for age, smoking, obesity, disease severity, site of onset, lifestyle, and other potential confounders. The adjusted HR was 1.38 (95% CI: 0.81–2.35, P trend =0.13) for fasting glucose concentrations ≥7.0 mmol/L vs <5.6 mmol/L. We did not observe any significant interactions between HbA1c levels and age, sex, smoking, body mass index, rate of disease progression of ALS, and site of onset (P-interactions >0.05 for all).

**Conclusion:**

In this prospective study, we observed that individuals with higher HbA1c levels at the baseline had higher risk of mortality, which is independent of other known risk factors.

## Background

Amyotrophic lateral sclerosis (ALS) is an incurable neurodegenerative disorder characterized by degeneration of both upper and lower motor neurons, with a median survival of 3–5 years after symptoms onset [1]. Several modifiable vascular factors and comorbidities have recently been studied as potential therapeutic targets for ALS. Interestingly, the presence of vascular risk factors (e.g., higher body mass index (BMI) and hypercholesterolemia) was reported to be associated with a lower risk and mortality of ALS [2–4].

In contrast, results regarding the association between diabetes mellitus (DM) and the risk, progression and survival of ALS were inconsistent or even contradictory [5–12]. DM was associated with a higher risk of ALS in a population-based cohort study from Taiwan [6] and a lower risk of ALS in studies conducted in western countries [7, 8]. Studies examined DM/ impaired glucose tolerance (IGT) status and ALS progression/survival failed to generate significant results [9–12]. However, some of these studies lacked information on obesity index and life style factors such as smoking that could confound the association between DM and ALS. Furthermore, status of DM and IGT was dominantly diagnosed by fasting blood glucose (FBG) concentrations in these studies, which only represented short-term glycemic status. Hemoglobin A1c (HbA1c) levels, which can reflect the cumulative glycemic history of the preceding 2 to 3 months, has recently been recommended as a possible substitute to FBG for the diagnosis of diabetes by the American Diabetes Association [13]. It provided a reliable measurement of chronic hyperglycemia and correlates well with the risk of developing long-term diabetes complications [13]. Elevated HbA1c levels have been regarded as an independent risk factor for cardiovascular diseases in individuals with or without diabetes [14]. However, the associations between HbA1c levels and the mortality and survival of ALS remain unknown.

Therefore, a large prospective study was conducted to examine the association between HbA1c and the mortality among 450 Chinese adults with ALS, adjusting for BMI, lipid profiles, disease status and lifestyle factors. We also explored potential interactions between HbA1c levels and these factors, in relation to ALS mortality. As a secondary exposure, the association between FBG concentrations and the mortality of ALS were also studied.

## Methods

### Participants and fellow-up

The study was conducted in the Department of Neurology, West China Hospital of Sichuan University in the southwest of China. Patients who were diagnosed with definite or probable ALS according to the El Escorial revised criteria [15] were recruited into our study at their first visit to our tertiary referral center from March 2009 to September 2014 (referred to as “baseline” in the current manuscript). Patients with progressive muscular atrophy, progressive bulbar paralysis, primary lateral sclerosis, and familial and juvenile ALS were excluded in the current analyses. There was a total of 558 sporadic ALS (SALS) patients met our inclusion criteria and 450 (80.6%) had complete hematological data. All 450 eligible patients were followed up with telephone or face-to-face interview in 3 or 6 months interval by neurologists (QQW, LYZ and YBH). Death information was collected from provincial public security bureau records and family reports. The institutional ethics committee of West China Hospital approved this study. All the participants were informed with the study and signed written informed consents. All methods were performed in accordance with the relevant guidelines and regulations.

### Assessment of HbA1c levels, FBG concentrations, and covariates

Blood samples after an overnight fasting (> 8 h) were collected at the baseline. HbA1c levels were assessed by ion exchange high-pressure liquid chromatography on a Tosoh G7 standard mode (Tosoh Corporation, Japan) using reagents according to the manufacturer’s instructions in the clinical laboratory, West China Hospital. Intralaboratory analytical coefficients of variation were <2%. Participants were categorized into three groups based on their HbA1c levels (<5.7% (38 mmol/mol), 5.7–6.4% (38–46 mmol/mol), and ≥6.5% (48 mmol/mol)) and the lowest group was used as the reference [16]. Participants who were treated with insulin or oral hypoglycemic agents were assigned into the group of HbA1c ≥6.5% regardless their actual HbA1c values.

FBG concentrations were measured with the hexokinase/glucose-6-pgosphate dehydrogenase method. The coefficient of variation using blind quality control specimens was <2.0%. Participants were identified as having DM if they had a FBG ≥7.0 mmol/L, or were treated with insulin or oral hypoglycemic agents [17]. And IGT was defined as FBG concentration between 5.6 and 6.9 mmol/L.

### Assessment of potential covariates

Baseline concentrations of fasting serum total cholesterol (TC), and triglyceride (TG) were measured by an enzymatic colorimetric method using an automatic analyzer (Olympus AU400; Olympus, Japan). Automatic hematology analyzer (Sysmex XE 5000, Kobe, Japan) completed blood counts. It performs hematology analysis according to the sheath flow DC detection method for erythrocytes and platelets and flow cytometry method using a semiconductor laser and fluorescent measurement for leukocytes and differential. Body height was measured, without wearing shoes, with an accuracy of 0.5 cm, using a stadiometer at the baseline. Body weight was measured to the nearest 0.1 kg with a steelyard scale, with underwear and no shoes. BMI was calculated as weight in kilograms divided by height in meters squared, and it was categorized according to the World Health Organization [18]. Blood pressure was also measured at the baseline and hypertension was defined as systolic BP ≥140 mmHg or diastolic ≥90 mmHg or use of antihypertensive medications in past 2 weeks. Information on lifestyle and personal history, including cigarette smoking status and drinking status, were collected via questionnaires [19]. Our previous studies have used the Chinese version of Addenbrooke’s Cognitive Examination-revised (ACE-R) and the Chinese version of the frontal assessment battery (FAB) to evaluate cognitive function and frontal lobe function in sporadic ALS patients and healthy controls [20, 21]. The ACE-R score less than 75 was defined as cognitive impairment and FAB score less than 16 was defined as frontal lobe dysfunction according to the previous studies [20, 21].

All the clinical data were collected at the baseline and during the follow-up evaluations, including age of onset, the region of symptom onset (upper limb, lower limb or bulbar), disease duration at baseline, diagnostic delay, the ALS Functional Rating Scale-Revised (ALSFRS-R) score, and the use of riluzole, gastrostomy percutaneous endoscopy (PEG) and noninvasive positive pressure ventilation (NIPPV). The rate of disease progression was assessed by the changes of ALSFRS-R per month (Formula: (48 – ALSFRS-R score at the baseline visit)/ month intervals between first symptom onset and the baseline visit).

### Statistical analysis

Person-years for each participant were calculated from the date of recruitment to the date of death or tracheotomy which was taken as equivalent to death, or September 1st, 2015, whichever came first. Continuous variables were expressed as the mean ± standard deviation (SD). The Student’s t tests were performed for between-group comparisons of continuous variables. Chi-Square tests were used to determine the differences of categorical variables between groups. The multivariate Cox proportional hazards regression model was performed to calculate hazard ratios (HRs) and their 95% confidence intervals (CIs) of ALS mortality across the exposure (i.e., HbA1c and FBG) categories as the proportional hazards assumption was satisfied. We adjusted for potential confounders, including age, sex, site of onset, rate of disease progression, smoking, alcohol drinking, BMI, TC concentrations, anemia, hypertension, and use of riluzole, NIPPV and PEG (because the treatments were associated with ALS mortality, as suggested in previous studies [22, 23]. Tests for linear trend in effect across HbA1c and FBG categories were performed using the median value in each category and treating this value as continuous variables. Because the use of hypoglycemic agents impacted levels of HbA1c and FBG, we conducted a sensitivity analysis by excluding individuals who used any hypoglycemic agents. We also tested the interactions between HbA1c levels and age, sex, BMI, anemia, hypertension, smoking status, site of onset and the rate of disease progression on ALS mortality. All the data were analyzed using SPSS18.0 statistical software (SPSS Inc., Chicago, IL, USA). A *P*-value less than 0.05 were considered as statistical significance.

## Results

### Clinical features

The mean age of enrolled patients was 55.4 ± 13.9 years, including 254 (56.4%) men and 196 (43.6%) women. The mean age of onset was 54.5 ± 12.2 years. The mean diagnostic delay was 15.7 ± 15.3 months. The ALSFRS-R total score was 39.1 ± 6.0 at the baseline (ranging from 12 to 48). For the site of onset, 244 (54.2%) patients had upper limb onset, 108 (24.0%) patients had lower limb onset, and 98 (21.8%) patients had bulbar onset. The average rate of disease progression was 0.77.

Demographic and clinical characteristics of ALS patients among different HbA1c status are shown in Table 1. Compared with ALS patients with lower HbA1c levels, those with higher HbA1c levels were more likely to be older. The results of other potential covariates in regards to HbA1c levels are reported in Table 2. Compared with ALS patients with lower HbA1c levels, those with higher HbA1c levels were more likely to have higher blood cell counts of erythrocytes, platelets, total leukocytes, neutrophils, lymphocytes, and monocytes, and blood concentrations of TG and TC. Other parameters, such as the frequencies of cognitive deficits and frontal lobe dysfunction, and the history of smoking and drinking, were not significantly associated with HbA1c levels. We did not find significant association between HbA1C levels and ALS severity --- HbA1c levels were similar when compared participants with low and high ALSFRS-R scores, based on median value (data not shown).

**Table 1:** Demographic and Clinical Characteristics of ALS patients in different HbA1c status (*N* = 450)

HbA1c, %	<5.7	5.7–6.4	≥6.5	P-trend
Number	263	152	35	
Age of onset, years	52.7(12.6)	57.0(11.2)	57.7(10.0)	<0.001
Age, years	53.1(15.3)	58.5(11.2)	59.4(9.8)	<0.001
Gender				
Male, %	57.4	53.3	62.9	0.52
Female, %	42.6	46.7	37.1	
Onset form				
Upper limb, %	54.8	51.9	62.9	0.20
Lower limb, %	20.9	29.6	22.9	
Bulbar, %	24.3	19.1	14.3	
ALS disease duration, months	16.0(15.5)	17.7(16.9)	19.7(16.4)	0.33
ALS diagnostic delay, months	14.9(14.8)	16.2(15.9)	19.1(16.6)	0.29
Education, years	7.9(4.3)	7.6(4.5)	6.2(2.9)	0.28
ALSFRS-R total score	39.3(5.7)	38.6(6.1)	38.3(7.5)	0.35
Rate of disease progression	0.75(0.65)	0.78(0.65)	0.84(0.71)	0.79
FAB (*N* = 195)				
<16 score, %	37.7	35.1	54.5	0.48
≥ 16 score, %	62.3	64.9	45.5	
ACE-R (N = 195)				
<75, %	38.0	40.7	33.3	0.86
≥ 75, %	62.0	59.3	66.7	

*Abbreviations*: *ALS* amyotrophic lateral sclerosis, *HbA1c* Hemoglobin A1c, *ALSFRS-R* amyotrophic lateral sclerosis functional rating scale-revised, *FAB* frontal assessment battery, *ACE-R* Addenbrooke’s Cognitive Examination-revised

**Table 2:** Results of other potential covariates in ALS patients between different HbA1c status (*N* = 450)

HbA1c, %	<5.7	5.7–6.4	≥6.5	P-trend
Number	263	152	35	
BMI, kg/m^2^	22.3(3.3)	22.0(3.0)	22.7(3.1)	0.57
BMI status				
Normal, %	84.8	86.2	82.9	0.79
Overweight, %	14.4	13.8	17.1	
Obesity, %	0.8	0.0	0.0	
Smoking, %	32.3	29.6	31.4	0.85
Alcohol drinking, %	32.7	26.3	25.7	0.33
Hypertension, %	11.8	19.7	17.1	0.08
Anemia, %	7.2	5.3	8.6	0.67
Erythrocytes, 10^^12^/L	4.5(0.5)	4.6(0.5)	4.7(0.5)	0.003
Platelets, 10^^9^/L	164.1(54.9)	171.8(55.9)	193.6(58.0)	0.01
Total leukocytes, 10^^9^/L	5.6(1.4)	5.9(2.0)	7.0(2.9)	<0.001
Neutrophils, 10^^9^/L	3.4(1.2)	3.7(1.8)	4.5(2.2)	0.001
Lymphocytes, 10^^9^/L	1.6(0.5)	1.7(0.5)	2.0(0.8)	0.001
Monocytes, 10^^9^/L	0.3(0.1)	0.4(0.1)	0.4(0.2)	<0.001
Triglyceride, mmol/L	1.4(0.9)	1.4(0.7)	2.2(1.8)	<0.001
Total Cholesterol, mmol/L	4.5(0.8)	4.8(0.9)	5.0(1.2)	<0.001
Use of hypoglycemic agents, %	0.0	0.0	60.0	<0.001

*Abbreviations: ALS* amyotrophic lateral sclerosis, *HbA1c* Hemoglobin A1c, *BMI* body mass index

Some patients also underwent an extensive genetic assessment using standard procedures. Four patients (2.07%, 4/193) had *SOD1* mutations, 3 patients (0.95%, 3/316) carried a *C9ORF72* GGGGCC repeat expansion, 1 patient carried a *FUS* mutation (0.47%, 1/212), 1 patient (0.53%, 1/187) carried a *CHCHD10* mutation, and no patient had *TARDBP* mutation (0/165). The frequencies of these ALS causative genes mutations were very low.

### Multivariate Cox proportional hazards regression model

**Fig. 1:**
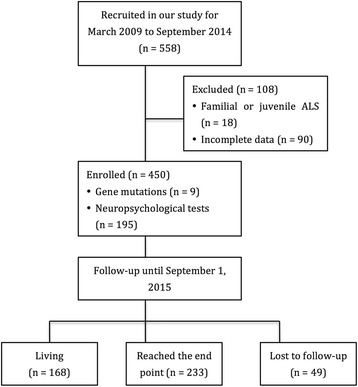
Flow chart for our study (ALS, amyotrophic lateral sclerosis)

**Fig. 2:**
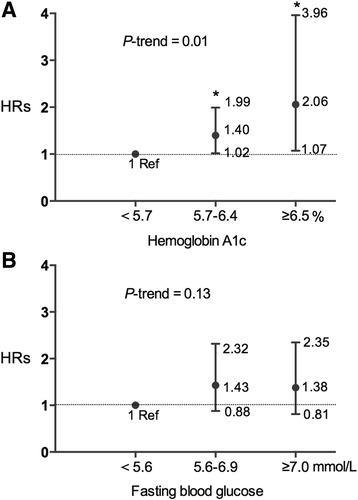
Hazard ratios (HRs) and 95% confidence intervals (CIs) for mortality according to Hemoglobin A1c (HbA1c, Panel **a**) and fasting blood glucose (Panel **b**) status in 450 individuals with ALS, adjusted for age, sex, site of onset, disease duration, ALS Functional Rating Scale-Revised score, smoking and drinking status, body mass index, total cholesterol concentrations, and use of riluzole, gastrostomy percutaneous endoscopy, and noninvasive positive pressure ventilation. * *P* < 0.05, relative to those with normal HbA1c, Error bars indicate 95% CI

During average 19.2 months of follow-up, 223 participants were deceased (Fig. 1). Survival time was not significant different between patients with limb onset and with bulbar onset (*P* = 0.20, HR =1.23), between patients with and without cognitive deficits (*P* = 0.88, HR = 0.97), and between patients with and without frontal lobe dysfunction (*P* = 0.79, HR = 0.95). As expected, higher BMI was associated with lower risks of mortality (adjusted HR =0.92 for each kg/m^2^ increment; 95% CI: 0.86–0.99; P trend =0.02) after adjustment for potential confounders. In contrast, higher baseline HbA1c levels were associated with a higher risk of mortality during the follow-up (Fig. 2a). The adjusted HR was 1.40 (95% CI: 1.02–1.99) for HbA1c of 5.7–6.4%, and 2.06 (95% CI: 1.07–3.96) for HbA1c ≥6.5%, relative to HbA1c <5.7% after adjustment for age, sex, site of onset, the rate of disease progression, BMI and other covariates. Each additional increased unit (%) of HbA1c was associated with a 50% (95% CI: 9%–107%, P trend =0.01) increased risk of mortality. We did not observe any significant interactions between HbA1c and other variables, in relation to survival of ALS patients (P-interaction >0.05 for all). In contrast, although there was a similar trend between higher FBG concentrations and a higher mortality risk, the association was not statistically significant (adjusted HR =1.37 for each mmol/L increment; P trend =0.13; Fig. 2b).

### Sensitivity analyses

Sensitivity analyses generated similar results. After we excluded 21 participants who were using hypoglycemic agents, higher baseline HbA1c levels, but not FBG concentrations were associated with a higher risk of mortality. (Adjusted HR =1.43 for each unit increment, P trend =0.02 vs. adjusted HR =1.29 for each unit increment, P trend =0.12, respectively). Excluding participants who carried *SOD1, C9ORF72, FUS,* or *CHCHD10* mutations did not change the results materially (data not shown).

## Discussion

In this prospective study, we found that the risk of mortality was doubled for individuals with HbA1c ≥6.5% at the baseline, compared to those with normal HbA1c level (<5.7%). Pre-diabetes status, as defined by HbA1c of 5.7–6.4%, was also significantly associated with higher risk of mortality. The significant association between HbA1c and ALS mortality was independent of known risk factors, including BMI, blood cholesterol concentrations, and disease severity. Interestingly, no significant association was found between FBG and ALS mortality.

A previous systematic review including 5 case-control and 2 cross-sectional studies [10] and a following pooled analysis including 6 ALS clinical trials [9] suggested that a history of pre-morbid DM2 was not an independent prognostic predictor for ALS progression and survival. It is worth noting that, all studies included in this review, except for one (conducted during 2006–2012) [24], were conducted in or prior to 2010 when HbA1c was recommended to be included in the DM diagnosis criteria [25].

Consistent with previous study [26], FBG was not associated with ALS mortality in our cohort. The FBG test is an excellent test for “in the moment” glucose levels, but it provided limited information about the time-course trend of the glucose levels. Analysis of HbA1c in blood provides evidence about an individual’s average blood glucose levels during the previous 2 to 3 months and has been recommended as a standard of testing and monitoring DM recently [13, 25]. There is a direct association between HbA1c and insulin resistance, where HbA1c has been shown to be more strongly associated with the insulin sensitivity in healthy individuals with normal glucose tolerance [27]. The value of HbA1c was found to have minimal overlap between subjects with normal glucose tolerances and subjects with DM2 when studying on glycemic spectrum for insulin resistance. Therefore, HbA1c is a reliable biomarker of insulin resistance for testing individuals for DM and pre-diabetes [28].

Several potential mechanistic pathways including mitochondrial dysfunction, oxidative stress, and insulin resistance could underlie the observed relation between HbA1c and the mortality of ALS. On the one hand, mitochondria, which plays a crucial role in cell apoptosis, has shown to be an early target in ALS pathogenesis and contribute to disease progression [29] and its dysfunction has a critical role in the pathogenesis of mutant superoxide dismutase 1 (SOD1) mediated familial ALS. On the other hand, previous studies have demonstrated a mitochondrial function defect in substrate oxidation with a decrease in mitochondrial density in disorders of insulin resistance [30]. And as a result of fuel oxidation, mitochondria generate considerable amounts of reactive oxygen species, which are implicated in the pathophysiology of DM and its complications [31]. Besides, oxidative stress is also associated with both insulin resistance [32] and motor neuron degeneration in ALS patients [33]. These studies suggest a potential link between the HbA1c, oxidative stress, mitochondrial dysfunction of insulin resistance, and the pathogenesis of ALS.

Systematic review study have demonstrated that the presence of iron deficiency with or without anemia (IDA) can lead to an increase in HbA1c values compared with controls, with no concomitant rise in glucose indices [34], which means HbA1c is likely to show a spurious increase due to iron deficiency and IDA. And, non-iron deficiency forms of anemia may lead to a decreased HbA1c [34]. This may lead to confusion when diagnosing DM using HbA1c. But in our study, no significant interaction was found between HbA1c and anemia (P-interaction >0.05). Anemia had no associated with the risk of mortality in ALS after adjustment in the Cox model without enough information on the iron levels. So, it clearly identifies the need for further investigation, especially in focusing on the associations between types and degrees of anemia, HbA1c and ALS. Although it is suggested that respiratory insufficiency that occurs with loss of respiratory muscle function may influence HbA1c values, the mean scores of ALSFRS-R subscale (respiratory function) were no significant difference in the subgroups regarding to the HbA1c values.

Our current study was included in a Chinese population to find out that higher baseline HbA1c levels but not FBG concentrations were associated with a higher risk of mortality. Interestingly, there were some previous studies exploring DM and the risk of ALS in different ethnicity generated conflicting results: in Europe-based studies, presence of DM was associated with a lower risk of developing ALS [8, 35], but in Asia-based study [6], participants with DM had a higher risk of developing ALS, relative to those without DM [5]. Previous studies reported DM associated with a higher risk of ALS in younger individuals [8, 36]. The inconsistent findings could be partially explained by the various confounders and ethnic variations across these studies. Firstly, Chinese ALS patients appeared to have a younger age at onset compared to Caucasians, as suggested by these studies [5, 6], Consistently, the mean age of onset in our current study was 54.5 years, relative to 65–68 years in aforementioned studies in Europe. Second, with different ethnic background, the impact of genetic factors on ALS might be different. For example, nearly 20% of familial Caucasian ALS patients had the repeat expansions in chromosome 9 open reading frame 72 (C9orf72) gene, but the prevalence of the expansions was very low or absent in Chinese ALS patients [37] and patients from Japan and Iran [38–40]. Further, Asian populations are prone to insulin resistant states at much lower BMI categories than Caucasian counterparts [41]. In our previous study based on 100,000 Chinese adults, we found that even individuals traditionally considered to be “thin” or “non-overweight” (BMI <20 kg/m^2^ or <23 kg/m^2^, respectively) exhibited steady increases in fasting blood glucose over time.

Several limitations of the present study should be noted. First, all the participants were solely recruited through a tertiary referral center in China; thus our results may not be generalized to other populations. However, the mean ALSFRS-R scores were similar (39.0 ± 6.0) between the current study and previous studies on this topic (39.0 ± 5.7) [9]. Second, we did not collect information on diabetes duration. A recent case-control study reported that DM duration might modify the association between DM and the risk of ALS [35]. Further exclusion of participants who used hypoglycemic agents (a surrogate for a longer DM duration) did not change results materially. Consistently, pre-diabetes, as suggested by HbA1c of 5.7–6.4%, was also significantly associated with higher ALS mortality. Finally, not all of enrolled patients in the current study were screened all of ALS causative genes mutations. This was one of the limitations of the current study.

## Conclusions

In this Chinese ALS cohort, we observed a strong dose-response relation between higher baseline HbA1c levels and higher future risks of mortality. Further studies performed in ALS patients with different ethnic backgrounds are needed to verify, whether higher HbA1c levels are also associated with increased ALS risks.

## Abbreviations

ALS: Amyotrophic lateral sclerosis
ALSFRS-R: ALS Functional rating scale-revised
BMI: Body mass index
C9orf72: Chromosome 9 open reading frame 72
CIs: Confidence intervals
DM: Diabetes mellitus
FBG: Fasting blood glucose
HbA1c: Hemoglobin A1c
HRs: Hazard ratios
IDA: Iron deficiency anemia
IGT: Impaired glucose tolerance
NIPPV: Noninvasive positive pressure ventilation
PEG: Percutaneous endoscopy gastrostomy
SD: Standard deviation
SOD1: Superoxide dismutase 1
TC: Total cholesterol
TG: Triglyceride
